# Potential Alterations of Functional Connectivity Analysis in the Patients with Chronic Prostatitis/Chronic Pelvic Pain Syndrome

**DOI:** 10.1155/2021/6690414

**Published:** 2021-05-07

**Authors:** Shengyang Ge, Qingfeng Hu, Yijun Guo, Ke Xu, Guowei Xia, Chuanyu Sun

**Affiliations:** ^1^Department of Urology, Huashan Hospital, Fudan University, 12 Central Urumqi Rd, Shanghai 200040, China; ^2^Department of Urology, Jing'an District Center Hospital of Shanghai, 259 Xikang Rd, Shanghai 200040, China

## Abstract

**Background:**

Chronic prostatitis/chronic pelvic pain syndrome (CP/CPPS) is one of the most common diseases in urology, but its pathogenesis remains unclear. As a kind of chronic pain which the patients suffered for more than 3 months, we investigated the influence on patients' brain functional connectivity in resting state.

**Methods:**

We recruited a cohort of 18 right-handed male patients with CP/CPPS and 21 healthy male right-handed age-matched controls. Their resting-state fMRI data and structural MRI data were preprocessed and processed by RESTPlus V1.22. To assess the integrity of the default mode network (DMN), we utilized the voxel-wised analysis that we set medial prefrontal cortex (mPFC) and posterior cingulate gyrus (PCC) as seed points to compare the global functional connectivity (FC) strength.

**Results:**

Compared with healthy control, the FC strength between left mPFC and posterior DMN decreased in the group of CP/CPPS (*P* < 0.05, GFR correction, voxel *P* < 0.01, cluster *P* < 0.05), and the FC strength between the left anterior cerebellar lobe and posterior DMN increased (*P* < 0.05, GFR correction, voxel *P* < 0.01, cluster *P* < 0.05). In the patient group, there was a positive correlation between the increased FC strength and the score of the Hospital Anxiety and Depression Scale (HADS) anxiety subscale (*r* = 0.5509, *P* = 0.0178) in the left anterior cerebellar lobe, a negative correlation between the decreased FC strength and the score of the National Institutes of Health Chronic Prostatitis Symptom Index (*r* = −0.6281, *P* = 0.0053) in the area of left mPFC, and a negative correlation between the decreased FC strength and the score of HADS anxiety subscale (*r* = −0.5252, *P* = 0.0252).

**Conclusion:**

Patients with CP/CPPS had alterations in brain function, which consisted of the default mode network's compromised integrity. These alterations might play a crucial role in the pathogenesis and development of CP/CPPS.

## 1. Introduction

In the urological clinic, chronic prostatitis/chronic pelvic pain syndrome (CP/CPPS) occurs in 5% to 10% of the male population [1]. According to the category of National Institutes of Health, CP/CPPS was defined as urological pain or discomfort in the pelvic region sustaining for no less than 3 months during the preceding 6 months that is associated with lower urinary symptoms and not in consort with a urinary tract bacterial infection [2]. Also, it is regularly connected with negative cognitive, sexual, behavioral, or emotional consequences [3].

The pathogenesis of CP/CPPS is still unidentified. The former studies suggested a specific role for immunological, neurological, endocrine, and psychological factors [4]. About 78% of the men with CP/CPPS are found to be fighting with psychological stress [5]. CP/CPPS is a kind of chronic pain disorder. Thus, CP/CPPS should be recognized as the compound, including a combination of spontaneous visceral and referred somatic pain characteristics (i.e., pelvic visceral and referred perineal pain) and the involvement of central sensitization in the spinal cord and brain [6].

Functional magnetic resonance imaging (fMRI) and higher magnetic field strengths could enable scientists to investigate the brain accurately and noninvasively. Based on hemodynamics, resting state in fMRI can assess functional connectivity reflecting synchronous ultraslow frequency oscillation between brain areas, which provides insight into how brain areas work together to produce pain and how these networks may be modified due to chronic pain [7]. The effect of blood oxygen level-dependent (BOLD) originates from an increase in metabolic activity and blood flow in activating parts of the central nervous system [8]. Functional connectivity (FC) describes the temporal synchrony or interregional cooperation between two or more spatially separate regions [9]. Previous studies showed FC between the motor cortex and the posterior insula may be among the most significant markers of altered brain function in men with CP/CPPS and may represent changes in the integration of viscerosensory and motor processing [10]. However, the extent of other FC changes in CP/CPPS and how deep the RSN reorganization would be specific/standard across this particular disease remains mostly unclear.

Many patients with CP/CPPS complained that the disease affected their life when they were in a state of relaxation and resting instead of concentrating, working, and physical exercise. Coincidentally, the default mode network (DMN), which was initially identified as the “task-negative network,” was originally recognized as domains that consistently showed synchronized deactivation during tasks and prominent activation in the resting state [11]. The DMN, based on the medial prefrontal cortex (mPFC) and posterior cingulate gyrus (PCC), exhibits higher metabolic activity at rest than during the performance of externally oriented cognitive tasks [12].

We hypothesized that due to chronic pain, the long-term hemodynamic changes in the brain that endure CP/CPPS do have diverse brain activity regions distinguished by resting-state fMRI. To figure out the profound mechanism of CP/CPPS, we utilized the BOLD fMRI method to investigate the FC alterations of CP/CPPS in patients with spontaneous pelvic pain during resting state.

## 2. Materials and Methods

### 2.1. Characteristics of Participants

The Ethics Committee approved the Jingan District Centre Hospital data collection, Shanghai (Ethical Approval Code: no. 2020-05). We collected data from 18 right-handed male patients with CP/CPPS and 21 healthy right-handed age and gender-matched controls (Table 1). To reduce the potential impact of senile brain atrophy, aged from 20 to 50 years male volunteers were required. Consistent with the definition of CP/CPPS, this disease was diagnosed by exclusion. The diagnosis was based on the chief complaint, medical history, routine urine, prostatic fluid examination, prostate ultrasound, and urinary system ultrasound to exclude acute or chronic bacterial prostatitis, prostate cancer, benign prostate hyperplasia, and other related diseases among the pelvic. All the patients had not taken any medications before, and they denied the other discomforts. The participants would be excluded if they got any acute or chronic infectious disease, other chronic pain diseases, internal organic diseases, history of malignant tumors, and chronic diseases that might contribute to peripheral nerve injury like diabetes mellitus and hypertension. We instantly took them to get the fMRI scan after their diagnosis through standardized clinical procedures, so all the patients of CP/CPPS were performed MRI examination only when they showed symptoms. Since we considered some clinical symptoms of the CP/CPPS involved in the lower urinary tract symptoms, all the participants were required to empty their urine bladders before entering the fMRI. After obtaining the written informed consent, all of the participants were requested to finish one National Institutes of Health Chronic Prostatitis Symptom Index (NIH-CPSI) scale [13] and one Hospital Anxiety and Depression Scale (HADS) [14]. The numerical rating scale (NRS) score was used to measure patients' spontaneous pain and eliminated potential pain patients in healthy control [15]. Then, we instructed the patients to take regular drug therapy according to their symptoms in case of delay in diagnosis. The acquisition, processing, and analysis of fMRI data were evaluated by a senior neuroradiologist together with our urologists.

### 2.2. Resting-State fMRI Data Acquisition

**Figure 1:**
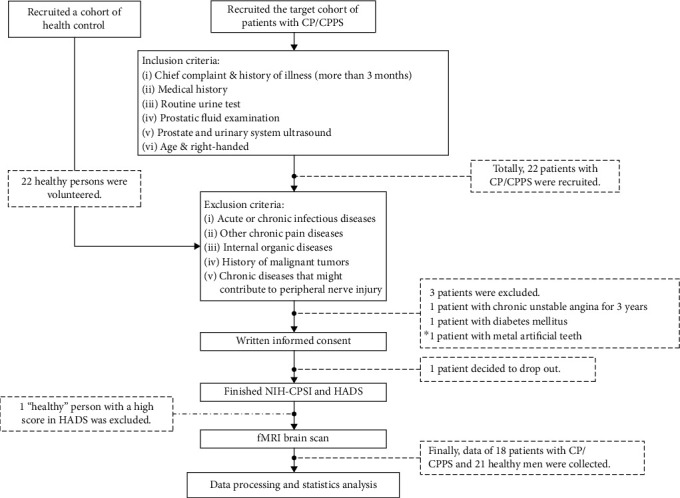
Study design flow chart. We initially recruited a cohort of health control (22 persons) and patients (22 persons). However, 1 person in health control and 4 persons in patient cohort were excluded. Their detailed reasons were demonstrated in the flow chart.

As shown in Figure 1, we primarily recruited a cohort of health control (22 persons) and patients (22 persons). After we eliminated unqualified candidates, fMRI data were obtained from 18 patients with chronic prostatitis/chronic pelvic pain syndrome rated spontaneous pain and also from 21 healthy control as participants inside the scanner. The resting-state fMRI data were obtained using a 3.0T GE MR750 MRI scanner with an eight-channel phased-array head coil at the Jingan District Centre Hospital, Shanghai. Whole resting-state fMRI data were acquired, using a gradient-recalled echo-planar imaging pulse sequence (repetition time (TR)/echo time (TE) = 2, 000/30 ms, FA = 90°, acquisition matrix = 64 × 64, field of view (FOV) = 22 × 22 cm^2^, slice thickness = 4 mm, no gap, 43 slices, and total 240 time points). The high resolution T1-weighted magnetic resonance images were collected by a three-dimensional fast spoiled gradient-echo dual-echo sequence (TR = 8100 ms, TE = 3.1 ms, FA = 8 deg, matrix = 256 × 256, FOV = 25.6 × 25.6 cm^2^, slice thickness = 1 mm, no gap, and 156 slices). To classify individual participants, the authors approached personal information during or after data collection. All the participants were required to keep their eyes closed, reduce thinking, maintain calm, and avoid falling asleep. In this step, the data would be eliminated if the senior neuroradiologist found any possible space-occupying lesion and vascular malformation in the brain.

### 2.3. Data Preprocessing Analysis

All the DICOM files in format were converted to NIFTI files by MRIconvert (http://lcni.uoregon.edu/jolinda/MRIConvert/). The resting-state fMRI data were also preprocessed using SPM12 (https://www.fil.ion.ucl.ac.uk/spm/) and RESTPlus V1.22 (http://www.restfmri.net). To avoid statistic errors, all the scans were examined before preprocessed. The preprocessing steps included as follows: (1) discarding the first 10 time points for reaching a steady-state magnetization and allowing all the participants to adapt to scanning noise, and the remaining 190 time points of image were processed in our following study; (2) slice timing correction, slice order could be Matlab formula: [1 : 2 : 43 2 : 2 : 42]; (3) head motion correction; (4) normalization, transforming the brain images to reduce the variability between individuals and allowing meaningful group analyses by using T1 image unified segmentation, with bounding box [-90, -126, -72; 90, 90, 108] and isotropic voxel size [3, 3, 3]; (5) spatial smoothing, by the convolution of the three-dimensional image with a three-dimensional Gaussian kernel with a full width at half maximum (FWHM) of 6 mm; (6) removing the linear trend of the time series caused by warming of the scanner or adaptation of the participants, with the time accumulation; and (7) nuisance covariable regression, including 6 head motion parameters, the cerebrospinal flow signals, and white matter signals. The mean value of the time series of each voxel was not added back in this step. After preprocessing in RESTPlus, the linear trend was removed. The fMRI data were temporally band-pass filtered (0.01-0.08 Hz) to decrease the very low-frequency drift and high-frequency noise from the cardiac and respiratory systems.

### 2.4. Voxel-Wised FC Analysis

FC analysis was performed using the RESTplus V1.22 after data preprocessing. Recognized as the core regions of the anterior and posterior DMN, the mPFC and the PCC were selected as seed points for analysis. Their Montreal Neurological Institute (MNI) coordinates are (-6, 52, -2) and (-8, -56, 26), respectively [16]. A sphere with a radius of 6 mm was selected as the region of interest (ROI). The average time series of all the voxels in ROI were extracted and calculated. Then, Pearson's correlation analysis was performed on the time series of each voxel in the whole brain. Thus, the functional connection diagram of mPFC and PCC with the whole brain was obtained. Pearson correlation analysis was performed on the time series of each voxel. The functional connection value was expressed by the correlation coefficient (*r* value), and then, it was converted to *Z* value by Fisher's to conform to the normal distribution.

### 2.5. Statistics Analysis

The results of characteristics of participants, NRS scores, NIH-CPSI scores, and HADS scores were compared using SPSS 17.0. A two-sample *t*-test was conducted based on the measurement data (age, duration of illness, NRS scores, NIH-CPSI scores, and HADS scores), and outcomes were listed as $\bar{\text{X}}\pm \text{S}$ (Table 1). A two-sample *t*-test was conducted based on the zFC values by the RESTplus software based on SPM12. Gaussian random field (GFR) theory, as multiple comparison correction, corrected functional connection values (voxel *P* < 0.01, cluster *P* < 0.05). The brain regions of increased or decreased FC values were taken as ROIs, and the values of each ROIs were extracted as independent variables by RESTplus. Pearson regression analysis was conducted with SPSS 11.0 as dependent variables to NIH-CPSI scores and HADS scores. The significant difference was set at *P* < 0.05. Activities larger than 5 voxels were displayed in pseudo-color on the calibrated standard brain map, and their MNI coordinates, positions, and voxel sizes of peak intensity were listed in a table (Table 2). The results of Pearson regression analysis were presented by GraphPad 5.0.

## 3. Results and Discussion

### 3.1. The Characteristics of Participants

We evaluated a male, dextromanual, and age-matched observational cohort (*P* = 0.0626). In the patient line (Table 1), the duration of the illness was about 20 months, accurately as defined. To avoid the abnormal results triggered by senile atrophy of the brain, we recruited volunteers aged between 20 and 50 years old. The score of NIH-CPSI revealed the patients suffered from CP/CPPS in the situation between moderate and severe edge, for a score above 18 means severe chronic prostatitis. Because the score of HADS would be meaningful when the score is more than 7, the main feature characteristic of patients in our study was anxiety as the score of HADS anxiety subscale was 8.11 ± 2.02 while the score of HADS depression subscale was 3.22 ± 0.79. Therefore, we paid our emphasis on the correlation between chronic pelvic pain caused by CP/CPPS and anxiety as a psychological symptom. The score of NRS was recognized as mild spontaneous pain that does not interfere with sleep.

### 3.2. The Abnormal Brain Regions by FC Analysis

**Figure 2:**
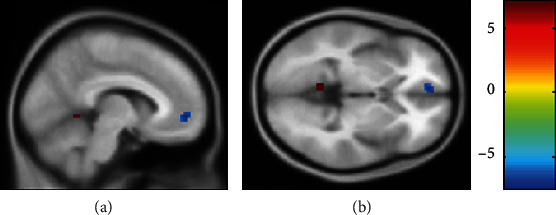
The differentiated brain regions with abnormal zFC values: (a) sagittal view; (b) axial view. The red region was positively activated, and the blue region was negatively activated (*P* < 0.05, voxel ≥ 5, GFR correction, voxel *P* < 0.01, cluster *P* < 0.05). Compared to the healthy control group, the patient cohort of CP/CPPS had differentiated brain regions with abnormal zFC values when we set the PCC as the seed point.

In our study (see in Figure 2 and Table 2), when we set the PCC as the seed point, we found the noticeably lower intensity of mPFC (peak intensity: -7.40) and the ascending intensity of posterior DMN regions like anterior lobe of cerebellum (peak intensity: 7.12). Simultaneously, although we also put the mPFC as the seed point, there was no statistical difference in zFC, which indicated that the anterior and posterior DMN showed a dissociation pattern.

### 3.3. The Correlation of Abnormal Brain Regions and Clinical Scale Scores

**Figure 3:**
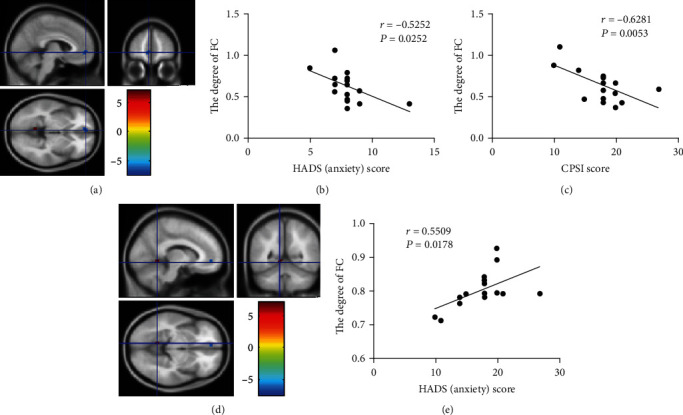
The differentiated brain regions with abnormal zFC values and their correlation with HADS anxiety subscale scores and NIH-CPSI scores. (a) Negatively activated clusters. (b) The correlation between the HADS (anxiety) score and the degree of FC in area of negatively activated clusters. (c) The correlation between the CPSI score and the degree of FC in area of negatively activated clusters. (d) Positively activated clusters. (e) The correlation between the HADS (anxiety) score and the degree of FC in area of positively activated clusters.

As shown in Figure 3, in the patient cohort of CP/CPPS, the zFC strength of the negative activated area in the left mPFC was negatively correlated with the score of NIH-CPSI scale (*r* = −0.6281, *P* = 0.0053). The zFC strength of negative activated area in the left mPFC was negatively correlated with the score of HADS anxiety subscale (*r* = −0.5252, *P* = 0.0252) while the zFC strength of positive activated area in the left cerebellum anterior lobe was positively correlated with the score of HADS anxiety subscale (*r* = 0.5509, *P* = 0.0178). In contrast, there was no statistical difference between the zFC strength of this area and the score of NIH-CPSI scale (*P* = 0.3821). Additionally, the correlation between the NRS scores and abnormal zFC strength revealed no statistical difference, which is possibly due to the limited sample size.

## 4. Discussion

The default mode network (DMN), identified as the “task-negative network,” was initially recognized as domains that consistently showed synchronized deactivation during tasks and prominent activation in the resting state [11]. According to the actual functional and anatomical classification of DMN, it is mainly subdivided into two parts, the anterior DMN and the posterior DMN, but both of these parts are responsible for spontaneous or self-generated cognition [17]. It has been proved that chronic pain is related to dysregulation within the DMN, and this dysregulation may help explain the mechanism of chronic pain syndromes [18, 19].

PCC is a part of the posterior medial cortex [20]. Its main functional characteristic in DMN is its central status [21]. PCC is a highly connected and metabolically active brain region, and its primary function is to integrate the central system, regulate the flow of information around the brain, and thus regulate attention and cognition to balance internal and external thinking [22]. The activity of PCC changes with arousal state and its interactions with other brain networks may be necessary for consciousness. The previous studies of fMRI indicated that a series of neurological and mental diseases like schizophrenia, autism, depression, and attention deficit hyperactivity disorder attribute to the abnormality of PCC [23, 24]. Chronic pain requires attention and competes for cognitive resource stimuli, so the internal functional alternatives in DMN might be the basis for the changes in attention and cognition during pain [16]. The previous study has proved that the FC strength within DMN of patients with chronic pain was connected to pain rumination that was a measure of an individual's constant attention to their pain and its potential negative consequences [25]. The internal changes of connectivity within the DMN may reduce the aptitude of other stimuli to attract the individual's attention away from the pain.

The mPFC plays an imperative role in both executive function and pain processing, and the mPFC undergoes a major reorganization in chronic pain [26]. Deactivation of mPFC output is causally correlated with both the cognitive and the sensory components of neuropathic pain [27]. The mPFC could serve dual, opposing roles in pain: (1) it mediates antinociceptive effects, due to its connections with other cortical areas as the main source of cortical afferents to the PAG for modulation of pain; (2) it could induce pain chronification via its corticostriatal projection, possibly depending on the level of dopamine receptor activation (or lack of) in the ventral tegmental area-nucleus accumbens reward pathway [28]. Painful stimuli may affect cognition—the mPFC and the mediodorsal nucleus of the thalamus form interconnected neural circuits that are important for spatial cognition and memory, and in chronic pain states, the mPFC is deactivated, and mPFC-dependent tasks such as attention and working memory are impaired [29]. Additionally, the functional and structural connectivity of mPFC-PAG might be associated with the individual differences in the tendency to self-concerns about pain [30]. So, due to the individual differences, it is possible to explain the phenomenon that there is no statistical difference between the patient group and the health control when we set the mPFC as the seed point, which may request to be further verified by subsequent large sample size experiments.

In the DMN, the changes of FC in the mPFC were also associated with self-reported anxiety levels [31]. The patients with chronic pain would not only feel anxiety but also get the fear related to the pain [32]. mPFC played a pivotal role in the brain circuits of fear, and the alternatives of the DMN might be the tractive force in this process. The fear related to the pain would promote the side effects of cognition and behavior [33]. More importantly, the fear of pain, which was motivated by the anticipation of pain, was a prognostic factor affecting the treatment of chronic pain [34]. Consequently, the clinical doctors must apprehend the strong impact of the cognitive process on the fear of pain, which even brings about avoidance behavior. In the clinic, many patients with CP/CPPS display poor compliance, i.e., they fail to take medicine on time and make psychological confrontation with the doctor. These patients should be carefully communicated and instructed. The main treatable processes conclude (1) to eliminate fear, explaining the idea “pain does not an equal injury”; (2) helping patients set realistic and reasonable expectations of treatment; (3) to improve self-efficacy and reduce self-catastrophizing, encouraging self-monitoring of patients; and (4) demanding the patients to the mental health department if the patients express a severe fear [35]. Otherwise, the anxiety, fear-avoidance, and density of chronic pain of the patients would aggravate each other. In similar studies, like chronic low back pain, complex regional pain syndrome, and knee osteoarthritis, there was decreased connectivity between mPFC and posterior DMN, and at the same time, there is increasing connectivity between mPFC and insular cortex, which is in proportion to pain intensity [18, 19]. These consequences were similar to the results in our study that there was decreased connectivity between mPFC and posterior DMN, and its zFC values negatively correlated to the prostatitis-like symptom scale scores, which also prompted that we should put emphasis on the alteration of the insular cortex in the patients with CP/CPPS in further study.

The cerebellum had anatomical connections to multiple regions of the frontal cortex and limbic region, critical for its involvement in visual and auditory processing, motion perception, cognition, and pain processing [36]. Yet, the specific function of the cerebellum in the process of pain and its role in pain disorders were still unclear [37]. The anterior cerebellum was also known as the old cerebellum, mainly involving motion control and body balance regulation [36]. Earlier studies had found anatomical connections between the cerebellum and the prefrontal cortex, which are also involved in motor function and somatic balance [36, 38]. A study had found that pain experiences were organized by somatization, and the cerebellum, activated by desomatization, indirectly accepted the information to engage in processing situational and emotional states partly through the cortex and subcortical connections [39]. During painful thermal stimulation, the bilateral frontal lobes of the cerebellum were active, which indicated the frontal lobes of the cerebellum might respond to motor control in response to painful stimuli after pain activation [39]. One study suggested that the cerebellum plays a vital role in the localizing of painful stimuli [40]. Because of the lack of relative evidence, it was speculated that the cerebellum is an integrator of multiple effector systems, including emotional processing, pain regulation, and sensorimotor processing [41, 42].

In this study, we speculated that the anterior cerebellum might respond to pain by motor control. Since the activated cerebellar motor regions were correlated with the anxiety levels in patients with CP/CPPS, it was also possible that multiple effector systems got involved in pain integration. There were still differentiated functional subregions in the anterior cerebellum lobe, which their precise mechanism of structure and function were unclear. Further investigation was required. In a study of chronic low back pain, the dysfunction of mPFC might lead to the possibility of unconscious pain behavior controlled by the cerebellum [43]. In another study of chronic migraine, it revealed that the cerebellum was not only involved in the pain process of migraine but also played a significant role in the prognosis of migraine [44]. There was increasing connectivity between the cerebellum and corpus callosum cortex, temporal lobe in the patients of chronic migraine, and decreasing connectivity between the cerebellum and prefrontal lobe, which correlated with the poor prognosis [44].

So, it illustrated that in addition to motor control, the frontal cerebellum might also affect the patient's mood through its emotional processing capacity or get through structural connections with the prefrontal lobe. It was also proved that the clinical treatment of CP/CPPS should take a measure of mind-body therapy [45, 46]. The aim of cognitive reappraisal to the patients was to reverse maladaptive cognition evaluation of their own [47, 48]. Through mindfulness meditation, there is a particular therapeutic effect as well [26, 49].

The degree of reorganization within DMN depends on the intensity and duration of chronic pain, mainly after more than 10 years of chronic pain [19, 50]. We hypothesized that as time went on, the functional connectivity of posterior DMN and mPFC would be decreased, and its connectivity to the anterior cerebellum lobe would be increased, which reflected the influence of the clinical symptoms of CP/CPPS gradually improved from emotion to more fear of pain. The fear of pain progressively changed the behavior of patients, while the avoidance would deepen the anxiety and distraction of DMN. Owing to the anatomical affiliation between the cerebellum and the prefrontal lobe, whether the anterior cerebellum and mPFC had the same or similar FC changes or structural reconstruction remained to be investigated in the future.

Consequently, patients with CP/CPPS had changes in brain function, which consisted of the compromised integrity of the default mode network. These changes may play a crucial role in the pathogenesis and development of CP/CPPS. Chronic pain should be best managed by using a multidisciplinary, biopsychosocial approach. With the development of the research on etiology and mechanisms of CP/CPPS, we would broaden our mind to expand our etiological treatment, provide new targets for therapeutic trials, and improve patients' quality of life.

Nevertheless, there might be some limitations in this research. The cohort of participants was relatively small. In the patients of CP/CPPS, the main alteration of emotion and psychology was anxiety, while they seldom revealed symptoms of depression, which may be because the duration of illness perhaps was a little shorter than other researches of chronic pain. This study was an observational study without intervention. Therefore, based on current findings, well-designed clinical therapeutic cohorts are needed to investigate further. Additionally, the tool fMRI could only describe macroscopic changes in the patients' brain function and structure. It has been proved that chronic pain may be related to neural plasticity, and the deeper cytological mechanism needs to be explored by other methods.

## 5. Conclusions

Patients with CP/CPPS had some alterations in functional connectivity of the brain, which mainly included the broken integrity of the default mode network. These changes may participate in the pathogenesis and progression of CP/CPPS.

## Figures and Tables

**Table 1:** The characteristics of participants.

	CP/CPPS patients (*n* = 18)	Healthy controls (*n* = 21)	*P* value
Gender	Male	Male	—
Age/years	30.92 ± 7.69	37.56 ± 7.86	0.0626
Duration of illness/months	20.00 ± 8.49	0	<0.001
NIH-CPSI score (1 + 2 + 3 + 4 + 5 + 6 + 7 + 8 + 9)	17.33 ± 4.40	0	<0.001
Pain and discomforts (1 + 2 + 3 + 4)	11.55 ± 2.16	0	<0.001
Lower urinary tract symptoms (5 + 6)	1.22 ± 0.78	0	<0.001
Impact on quality of life (7 + 8 + 9)	4.55 ± 2.63	0	<0.001
NRS (4)	2.44 ± 0.50	0	<0.001
Severity of symptoms (1 + 2 + 3 + 4 + 5 + 6)	12.77 ± 2.34	0	<0.001
HADS (anxiety)	8.11 ± 2.02	0.90 ± 1.22	<0.001
HADS (depression)	3.22 ± 0.79	0.33 ± 0.47	<0.001

**Table 2:** The details of differentiated FC in brain region.

Brain region	MNI coordinates			Voxel numbers	Peak intensity
	*X*	*Y*	*Z*		
Left cerebellum anterior lobe	-9	-51	-3	7	7.12
Left mPFC	-6	51	-3	13	-7.40

## Data Availability

The datasets used and analyzed in the current study are available from the corresponding author on reasonable request.
